# The Enigmatic Function of PARP1: From PARylation Activity to PAR Readers

**DOI:** 10.3390/cells8121625

**Published:** 2019-12-12

**Authors:** Tatiana Kamaletdinova, Zahra Fanaei-Kahrani, Zhao-Qi Wang

**Affiliations:** 1 Leibniz Institute on Aging–Fritz-Lipmann Institute (FLI), Beutenbergstrasse 11, 07745 Jena, Germany; Tatiana.Kamaletdinova@leibniz-fli.de (T.K.); Zahra.Fanaeikahrani@leibniz-fli.de (Z.F.-K.); 2 Faculty of Biological Sciences, Friedrich Schiller University Jena, 07743 Jena, Germany

**Keywords:** PARP1, PARylation activity, PAR, PAR-binding motif, PAR binder

## Abstract

Poly(ADP-ribosyl)ation (PARylation) is catalysed by poly(ADP-ribose) polymerases (PARPs, also known as ARTDs) and then rapidly removed by degrading enzymes. Poly(ADP-ribose) (PAR) is produced from PARylation and provides a delicate and spatiotemporal interaction scaffold for numerous target proteins. The PARylation system, consisting of PAR synthesizers and erasers and PAR itself and readers, plays diverse roles in the DNA damage response (DDR), DNA repair, transcription, replication, chromatin remodeling, metabolism, and cell death. Despite great efforts by scientists in biochemistry, cell and molecular biology, genetics, and pharmacology over the last five decades, the biology of PARPs and PARylation remains enigmatic. In this review, we summarize the current understanding of the biological function of PARP1 (ARTD1), the founding member of the PARP family, focusing on the inter-dependent or -independent nature of different functional domains of the PARP1 protein. We also discuss the readers of PAR, whose function may transduce signals and coordinate the cellular processes, which has recently emerged as a new research avenue for PARP biology. We aim to provide some perspective on how future research might disentangle the biology of PARylation by dissecting the structural and functional relationship of PARP1, a major effector of the PARPs family.

## 1. Introduction

Poly(ADP-ribosyl)ation (PARylation) is a concerted and dynamic process. Poly(ADP-ribose) polymerases (PARPs, also known as ARTDs) catalyze the transfer of the ADPr unit from NAD^+^ to form a long and branched chain of negatively charged poly(ADP-ribose) (PAR) on specific amino acid residues (e.g., glutamates (E), lysine (K), arginine (R), serine (S), and aspartate (D)), on PARP1 itself and other acceptor proteins [1,2]. Removal of PAR from its substrates is a rapid event which is regulated by PAR-degrading enzymes, such as PARG (poly ADP-ribose) glycohydrolase, ADP-ribose hydrolases (ARHs), macrodomain-containing ADP-ribose erasers, and ADP-ribosyl lyase [3,4,5].

**Figure 1:**
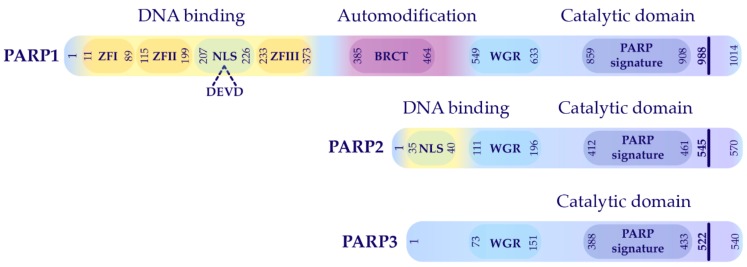
The scheme of PARP1, PARP2, and PARP3 structure and its functional domains. ZFI: zinc finger I, ZFII: zinc finger II, ZFIII: zinc finger III, NLS: nuclear localization signal, BRCT: BRCA1 C-terminal, DEVD: caspase cleavage site, and AAA: ankyrin repeat. The major domains and active catalytic sites are marked.

The PARP family includes ADP-ribosyltransferases which share sequence homology with each other. In the human genome, 18 PARP family members have been identified thus far. Among them, PARP1 is a founding member of this family, well-known for its essential function in DNA damage repair and other cellular processes, such as chromatin remodeling, transcription and cell death signaling [2,6,7]. In fact, the term “PARP family” is misleading, because, besides PARP1, only PARP2 and PARP3 as well as PARP4 and PARP5 (tankyrases 1 and 2) have been shown to harbor PARylating activity, although the nature of PAR generated by these enzymes is different [3,8]. PARP1 is believed to conduct more than 90% of total PARylation activity at least in response to DNA damage; all other PARPs have minor PARylation activity in response to DNA damage [9]. With respect to structure, PARP2 and PARP3 are closely related (Figure 1). While PARP2 has DNA damage inducing PARylation activity, PARP3 has no DNA binding domain. Functionally, PARP2 is believed to be major PAR-forming PARP after PARP1 and can compensate for the loss of PARP1 in vivo. This point is described in the relevant session below.

PARP1 was first described by its role in the repair of alkylating agent-induced DNA base damages and also single strand breaks (SSBs). PARP1 is involved in base excision repair (BER) via recruitment of BER scaffold protein XRCC1 (X-ray cross-complementing group 1) to the damage site. It has also been shown to play pivotal roles in the repair of DNA double-strand breaks (DSBs) via homologous recombination (HR), as well as nonhomologous end joining (NHEJ) [9]. PARP1 is also able to bind DSBs and modulates recruitment of DSB repair factors [10]. For example, it promotes PAR-dependent recruitment of meiotic recombination 11 (Mre11) during DNA end resection in HR. Also, by cross-talking with DNA–PKcs, PARP1 is involved in C-NHEJ [9]. Intriguingly, both PARP1 and PAR act as binding surfaces for numerous other proteins and regulate several cellular processes, such as DNA repair, transcription, cell death, chromatin remodeling, inflammation, metabolic regulation, cell cycle regulation, differentiation, proteasomal degradation, RNA processing, and modulation of tumor suppressor (see Reviews [6,7,8,11]). These studies highlight PARP1 as a multifaceted enzyme that modulates various sophisticated cellular processes, depending on cellular stressors, own interactors and its product PAR and, more importantly, under physiological conditions of a multiple cellular organism.

Our understanding of PARP1′s function in different cellular processes, including DNA damage response, has been advanced by a variety of biochemical, cellular, and animal model-based studies. In addition, chemical PARP inhibitors (that mainly target PARP1) have been used to study PARP function and further developed for medical purposes i.e., disease treatment. Despite intensive research on PARP1, many aspects of this enzyme remain poorly understood, which leaves many open questions. For example, which of the described functions are related to the PARP1 protein itself and which are attributed to its enzymatic product PAR [2]? Several recent reviews have comprehensively described the PARP family [7,12,13] and also summarized development of PARP inhibitors in clinical applications [11,14,15]. Hence, this review will concentrate on an overview of biochemical and biological studies of PARP1, aiming to illustrate the contribution of PARP1 protein and its enzymatic product PAR, as well as PAR interactors in biological processes in vivo.

## 2. The Biological Function of PARP1 and Its Enzymatic Activity

### 2.1. PARP1 Structure and Functional Domains

PARP1 is 1014 amino acids long with a total molecular weight of approximately 113 kDa. Structurally, this enzyme is composed of three main domains including an N-terminal DNA-binding domain (residues 1–353), an automodification domain (residues 389–643), and a catalytic domain in the C-terminal (CAT) (residues 662–1014). In the N-terminal domain, there are three zinc finger DNA-binding domains, namely ZnFI, ZFII, and ZFIII. ZFI and ZFII are required for recognition and binding to DNA damage sites, while Zn3 has an important role in the enzyme activation upon DNA binding. A nuclear localization sequence (NLS, sequence: KRK-X(11)–KKKSKK) is also located in the N-terminal region that leads PARP1 to the nucleus [16,17]. Worthy of mention is the presence of a specific caspase cleavage site at the position 211–214aa (DEVD), which is cleaved during apoptosis [18]. The central auto-modification domain is enriched in glutamate (E) and lysine (K) residues, which serve as the sites for PARylation. This domain is composed of a breast cancer type 1 susceptibility protein (BRCA1), C-terminal (BRCT) motif, and the WGR domain with several conserved tryptophan, glycine, and arginine residues. BRCT motif mediates protein–protein interactions, while the WGR domain interacts with ZFI, ZFIII, CAT, and the DNA and is important for DNA-dependent activity of the catalytic domain. The CAT domain contains a ‘‘PARP signature′′ sequence comprised of the NAD acceptor site and the residues contributing to the initiation, elongation, and branching of PAR [18] (Figure 1).

### 2.2. Functional Domain Mapping of PARP1

In general, PARP1 is located in the nucleus and is activated after binding to SSBs and also DSBs, to catalyze PARylation of itself (automodification) and of various acceptor proteins (heteromodification) [10]. It can also be activated by events other than DNA damage, such as distorted DNA structures, interaction with proteins like histones, changes in the level of metabolites or ions, and post-translational modifications [7,19], although the biochemical mechanisms are poorly understood. A series of biochemical and cell-based studies have been performed to decipher the structure and function of the PARP1 enzyme, as well as its PARylation activity. To this end, mutagenesis and deletion mutant studies have been employed to disclose the functional domains of this enzyme. The special characteristics of each are explained in the following.

To determine the contribution of each domain of PARP1 to DNA damage response, different structural units of the enzyme have been deleted or mutated and the functional consequences examined. For instance, the essential role of the DNA-binding domain (DBD) is integral for activation of the protein and its enzymatic activity [20,21]. Within this domain, three zinc fingers (ZF) are positioned at the N-terminus: ZFI (aa 1–111), ZFII (aa 117–201) and ZFIII (aa 279–333) [21]. Deletion of both ZFI and ZFII causes a marked decrease in DNA-binding affinity (over 250-fold) and loss of enzymatic activity [22,23]. ZFIII-deleted mutant is also enzymatically inactive because site-directed mutagenesis of the residues W318 and T316 in ZFIII abolishes DNA-dependent PARP1 activation [24]. In contrast, deletion of ZFII alone has no dramatic effect on the automodification activity; however, it causes a substantial reduction in the DNA-binding affinity. This suggests that the ZFI and ZFIII fingers of the enzyme are essential for the PARylation activity and ZFII is dispensable, but more related to the binding affinity toward DNA [23].

The introduction of several mutations located in the N-terminus, such as Q40A, F44A, D45A, V48A, and F44A/V48A impair auto-modification by PARylation [22]. F44A, V48A, and F44A/V48A mutants were deficient in the ability to bind DNA, whereas Q40A and D45A mutations do not affect DNA binding of the enzyme. Thus, the impact of these mutations on PARP1 activity is probably associated with their interactions with other domains, which are required for DNA-dependent activity of the enzyme [22]. Similarly, M43D and F44D replacements render a significant decrease of the recruitment of PARP1 towards DNA damage sites. The substitution of V144 and P149 in ZFII (V144E/P149D and V144E/P149I) causes a similar defect in binding to a DNA lesion [25]. Furthermore, PARP1 lacking the WGR domain (aa 525–656) was shown to be incapable of producing PAR, whereas the deletion of the BRCT domain bore no influence on auto-modification as well as PAR formation [26]. The automodification of PARP1 is dependent on serine residues, because mutation of serine residues, S499, S507, and S519 in the automodification domain abolishes HPF1 (histone PARylation factor 1)-dependent autoPARylation of PARP1 [27]. Taken together, these studies establish the concept that PARP1 enzymatic activity is solely dependent on the DNA binding by the N-terminal domains of PARP1.

Many studies have tried to map the enzymatic activation domains. Early efforts showed that the 40-kDa C-terminal part of the protein is an independent catalytic domain [28]. Particularly, K893 is critical to poly(ADP-ribose) polymerase activity [28]. The direct contribution of K893, as well as D993, is in the initiation of the poly(ADP-ribosy1)ation reaction, whereas D914 and K953 are only indirectly involved in PARP1 activity [29]. Furthermore, the conserved residue E988 plays an important role in the synthesis and elongation of PAR chains. Mutation of this amino acid to glutamine (Q) and/or alanine (A) produces a more than 2000-fold reduction in the polymer elongation [30]. Interestingly, this mutation has a minor impact on binding affinity of the enzyme to NAD^+^, indicating that the function of this residue is mostly related to catalytic activity [30]. A gain-of-function mutation of PARP1 (L713F) was shown to have over nine times higher catalytic activity than the wild-type enzyme, but the Michaelis constant *(Km*) for NAD^+^ and the size of the PAR chain were similar to those of the intact enzyme [31].

One of the fundamental studies performed to understand the structure–function relationship of PARP1 and the key residues involved in catalytic activity, was conducted using a library of human PARP mutants expressed in *Escherichia coli*, in which the mutations were mostly located in the catalytic domain [32]. Among these mutations, 26 individual substitutions all resulted in PARylation inactivation. These authors confirmed that E988 is necessary for the elongation of the PAR chain. The substitution of threonine (T) and tyrosine (Y) at positions 982–987 also led to the inactivation of PARP1 activity, demonstrating the key contribution of this region to its catalytic activity. Interestingly, mutations of several important amino acids (e.g., R847, E923, and G972) all resulted in less branched PAR [32].

In summary, numerous studies employing point mutation and deletion of mutant PARP1 demonstrate that the PARP1 protein can have various functions biochemically and biologically. A number of important mutations of PARP1 are summarized in Table 1.

### 2.3. Biological Functions of PARP1

Within the last few decades there has been much effort to dissect PARP1 biological function and its role in cellular processes. At the early stage of research, chemical inhibitors and NAD^+^ analogs, e.g., 3′-aminobenzamide (3-AB), against the enzyme were used intensively to test the function of PARP1, especially in DNA repair. Whilst progress has been made, these inhibitors are not efficient tools for gaining detailed information on the role of PARP1 in cellular responses to DNA insults, due in part to their side effects and interference with other pathways unrelated to PARP1 [33]. Nevertheless, specific PARP inhibitors have been developed for disease treatment in clinics [11,14,15,34,35]. The main knowledge about the biology of PARP1 and PARylation comes from mutagenesis studies of the enzyme using cellular and animal experimental systems.

The major breakthrough in delineation of the biological function of PARP1 was achieved through genome editing in mouse models, via the gene targeting technology in embryonic stem cells (ESCs). PARP knock-out mice have been generated by several laboratories [36]. Wang et al. first generated a PARP1 knock-out mouse line and showed that mice lacking PARP1 were surprisingly viable and fertile, given the essential function of PARP1 in DNA repair. These mutant mice displayed no visible abnormalities at birth, indicating that the enzyme, if deleted, is dispensable for embryogenesis and development. The authors showed that mouse embryonic fibroblasts (MEFs) derived from PARP1 null mice displayed negligible DNA repair defects [37]. These observations are compatible with the model proposed by T. Lindahl showing that PARP1 is a DNA nick sensor and can bind to DNA lesions. If it cannot be removed for example by auto-PARylation, it inhibits DNA repair and is toxic to cells; thereby known as a “trapping model”. Where PARP1 is not present, the major BER is not affected—or likely some other mechanism can substitute PARP1 [38,39]. Interestingly, PARP1^−/−^ mice are sensitive to acute radiation-induced toxicity of the small intestine [40,41]; whilst other studies show that PARP1^−/−^ cells have a hypersensitivity to cell death induced by alkylating agents [40,42]. Moreover, PARP1-deficient cells accumulate in G2/M phase after treatment with methylmethanesulfonate (MMS) [43]. The PARP1 knock-out mouse model has been used to study the role of PARP1 or PARylation in several biological processes including genomic stability, stress response and apoptosis [44,45]. A prominent phenotype of PARP knock-out mice and cells is a general decrease of genomic stability, characterized by an elevated rate of sister chromatid exchanges (SCEs) and an increased frequency of chromosome breaks, chromosome fusions, aneuploidy, and telomere shortening—demonstrating that this enzyme has a pivotal role in the maintenance of genome integrity, with or without genotoxic stress [41,46].

PARP1 and PARP2 can homo- and heterodimerize and PARylate each other. PARP2 also interacts with XRCC1 and other proteins involved in BER [3,8]. PARP2 knock-out mice develop normally but are hypersensitive to whole-body radiation [47]. PARP2 deficient cells show a delayed DNA repair after alkylating agent treatment. Mutant cells exhibit genomic instability, defective BER, and cell cycle progression [47]. PARP3 interacts with proteins involved in BER and NHEJ pathways, indicating its role in DNA repair. PARP3 acts synergistically with PARP1 in response to DNA damage. However, its deletion in mice does not cause such dramatic phenotype as PARP1 and/or PARP2 knock-out. PARP3 knock-out mice do not show obvious phenotypical abnormalities and exhibit normal response to whole-body radiation [48]. Although PARP1 knock-out mice are viable, double knock-out of PARP1 and PARP2 results in early embryonic lethality [47]. In contrast, PARP1/PARP3 double knock-out mice are viable, but they are hypersensitive to radiation [48]. These genetic studies have demonstrated a compensatory function of PARP2 in the viability of PARP1 knock-out mice. Likely, PARP1 and PARP2 are mainly responsible for PARylation. Thus, PARylation activity is essential for embryonic development.

Although the cleavage of PARP1 by caspases at its DEVD site is a common event during apoptosis, PARP1 null MEF cells show no obvious defect in cellular apoptosis upon treatment with dexamethasone and anti-Fas (for lymphoid cells), with tumor necrosis factor alpha (TNFα) and γ-irradiation as apoptotic agents [41]. PARP1 null cells reconstituted with caspase-resistant PARP1 (PARP1-D214N) have been shown to display increased apoptosis and necrosis after TNFα treatment [49,50,51], suggesting that the cleavage product, e.g., 24 kDa N-terminus of PARP1, irreversibly binding to DNA prevents DNA repair once apoptosis occurs. Another study, using PARP1^−/−^ MEFs ectopically expressing uncleavable PARP mutant (PARP1-D214A) exhibited a delay of cell death after CD95 stimulation, implying that the cleavage of PARP1 can influence the programmed cell death [42].

**Figure 2:**
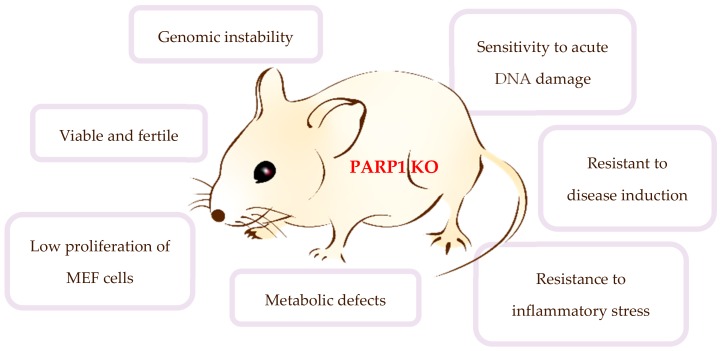
General characteristics of PARP1 knock-out (KO) mice. These mutant mice are surprisingly viable, fertile and have normal life, despite the genome being unstable. While these mice are extremely sensitive to alkylating agents and ionizing radiation, they are resistant to inflammatory stimuli, and ischemic and endotoxic treatment.

Another interesting feature of PARP1-deficient mice is their attenuated response to endotoxic shock, diabetic induction, stroke, and intestinal and renal ischemia-reperfusion [44,52,53]. These phenotypes are believed to be attributable to the function of PARP1 in NF-kB activation. The lack of PARP1 leads to a remarkable decrease in the release of inflammatory mediators downstream of NF-kB transcription factor [44,54]. Apart from being resistant to septic shock, PARP1 null mice show a certain degree of protection from other disease induction, such as ischemic stroke [55,56], traumatic brain injury [57], and streptozotocin-induced diabetes [58,59], indicating the role of PARP1 in the pathogenesis of these diseases. In contrast, in the case of the multiple sclerosis model, they were seen to develop more severe signs of the disease [60]. Of note, mice carrying a caspase-resistant PARP1 (PARP1-D214N knock-in mutant) did develop normally, showing no obvious phenotype [52]. However, these mice were resistant to endotoxic shock and intestinal and renal ischemia-reperfusions due to compromised inflammatory response. Non-cleaved PARP1 impairs NF-kB mediated transcription. These results suggest that the caspase-cleaved fragments of PARP1 assist transcription of inflammatory factors [52]. Furthermore, PARP1^−/−^ mice have shown metabolic defects which are strain-specific. For instance, in two different studies investigating the effect of PARP1 deficiency on metabolism and obesity, different results emanated from distinct mouse genetic backgrounds. Using C57BL/6J mice, Bai et al., found that PARP1^−/−^ mice have enhanced energy consumption but weigh less [61]. By contrast, PARP1^−/−^ mice in the 129/Sv background were susceptible to obesity, especially at older ages [37], and also prone to diet-induced obesity [62] (Figure 2). These diverse effects on different disease models show the significant functions of PARP1 in distinct signaling pathways.

The many studies on PARP1 knock-out mouse models have generated a great amount of information about this enzyme and its function in diverse cellular and disease processes; yet, the complexity of PAR and the PARylation modification irrespective of the PARP1 protein itself, often complicate the understanding of PARP biology. Attempts have been made to dissect the function of PARP1 as a protein from its enzymatic activity and activity product, PAR. The PARP1-E988K and PARP1-L713F mutant proteins were investigated through their overexpression in the TALEN-generated PARP1 knock-out HeLa cells. PARP1-E988K delayed its recruitment and persisted longer at the site of DNA lesions and induced G2 arrest in the cell cycle [63]. The gain-of-function mutant PARP1-L713F promoted cell apoptosis, even in the absence of DNA damage induction [63].

Furthermore, Schuhwerk et al. generated a PARP1 knock-in mouse model harboring a point mutation in the catalytic domain of PARP1 (D993A). This mutation was associated with decreased enzyme kinetics and the complexity of PAR chain designated as hypo-PARylation. Homozygous PARP1-D993A mutant mice showed no obvious phenotypic alterations, but were hypersensitive to alkylating agents and died six days after intraperitoneal injection of the MNU (*N*-methyl–*N*–nitroso urea); the wild-type control did not show any sign of disease. Despite a significant delay of PARylation after DNA damage, the homozygous PARP1-D993A mutant cells showed only a mild impairment of BER [64]. The conclusion drawn from this is that although neither one of PARP1 protein itself and its complete activity is necessary for the development, the kinetics of PARylation and the complexity of PAR are important for the final outcome of biological response to genotoxic stress in vivo [64].

In summary, studies employing biochemistry and cell and molecular biology, as well as mouse models, have generated a great amount of information regarding the molecular and cellular function of PARP1, demonstrating its multifaceted function in diverse cellular processes. The deletion of the entire PARP1 protein however cannot simply refer to the functions of PARP1—the most fascinating of which is that the lack of PARP1 in cells and mouse models leads to both negative (cytotoxic and genotoxic stress) and positive outcomes (protective inflammatory response) [40,44,45]. The nature of the PARP1 protein, with its different functional domains, each of which can be dependent or interdependent of each other, makes it difficult to reconcile these functional differences using PARP1 null mice and cells—a point further complicated by the fact that both PARP1 and its enzymatic product PAR have diverse interaction partners, which play diverse roles in many pathways within the cell.

## 3. PAR-Binding Motifs and PAR Reader Proteins

### 3.1. PARylation and PAR

**Figure 3:**
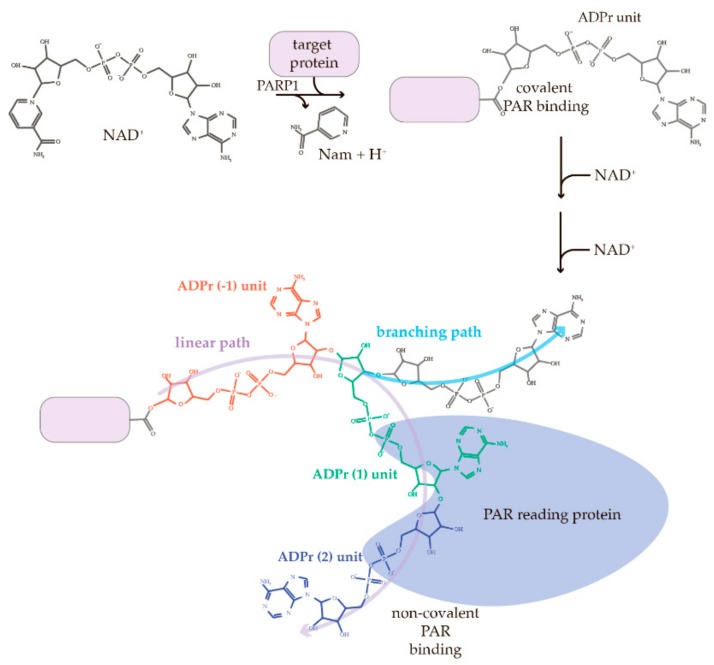
The scheme of PAR chain synthesis on the target protein. PARP1 cleaves the glycosidic bond between nicotinamide and ribose of NAD^+^, then provides the covalent attachment of ADP-ribose (ADPr) onto target proteins. Upcoming NAD^+^ molecules are used to further chain elongation via 2′,1′′-*O*-glycosidic bond. The branching point is 2′′,1′′′-*O*-glycosidic bond. PAR chains can be read by proteins containing specific and distinct PAR-binding motifs and bound non-covalently. The reading via the PBZ domain is illustrated in the picture.

PAR is a polymeric ADPr unit formed from NAD^+^ by PARPs (Figure 3) [65]. As a covalent attachment of PAR to proteins, the PAR formation and degradation after DNA damage, occurs very rapidly [38]. The addition of the first ADPr moiety to the protein occurs covalently to the D, E, and K residues via an ester bond [66]. The new unit of ADPr binds to the former unit via a 2′,1′′-*O*-glycosidic bond. A PAR chain may also form branched structures via a 2′′,1′′′–*O*–glycosidic bond [65] (Figure 3).

Proteins can also bind PAR non-covalently (Figure 3) through distinct and diverse PAR-binding motifs. The non-covalent PAR-binding to target proteins has different dissociation constants (*K_d_*), which may decide the interconnection to the adequate cellular responses. Due to the variability of PAR structures and the diversity of PAR-binding motifs presented in the target proteins, interaction of PAR with their target proteins seems to transmit certain signals or messages from one pathway to another during cellular response to extrinsic and intrinsic stressor, thus dictating the cell fate. Although the major, if not all, enzymes which catalyze initiation, formation, extension, and branching of PAR chains have been well studied, the precise conformation and nature of PAR are poorly defined; due in part to technical constraints, for example, in chemical measurement and synthesis of defined length, branching, or combination PAR. Therefore, study on the function of PAR–protein interactions has consisted mainly of searching and characterizing PAR-binding motifs of target proteins. Recognition of PAR by different proteins may depend on the structure of PAR and the binding motif of target proteins. This may also be influenced by the *K_d_* values, which vary from high (*K_d_* ≤ 10^−9^ M) or medium (10^−9^ M < *K_d_* ≤ 10^−6^ M), to low affinities (*K_d_* > 10^−6^ M) [67]. As such, it is plausible that PAR-binding by different target proteins/partners can serve an important signaling to downstream executers. PARP family members modify a huge amount of the proteins by poly and/or mono ADP-ribosylation—currently counted at about 2500 proteins [68]. PARPs put short or long, branched or linear, PAR to the diversity of the proteins to label them as “to do” marks. Here, our discussions focus on the studies of PAR readers (or binders) together with specific and distinct PAR-binding motifs.

### 3.2. PAR-Binding Motifs

PAR chains covalently attached to acceptor proteins provide a platform to search for their non-covalent binding partners. These binding partners read PAR by distinct PAR-binding motifs and determine the destiny of the PARylated targets or carry out their tasks in transmitting the message originating from the site where stimuli activate PARylation. Our focus here is confined to the PAR readers during DNA damage detection, signaling, and repair.

In the last 20 years or so, several types of PAR-binding domains have been identified (Table 2), among which PAR-binding zinc finger (PBZ), PAR-binding motif (PBM), and macrodomains are the most investigated [2,69]. While PBM binds the structure between the second phosphate of the ADPr (1) residue to the first phosphate of the next residue ADPr (2), the PBZ domain does similar, but also the ADPr residue of the previous ADPr (−1) unit [65]. Macrodomains completely bind ADPr residue of the PAR chain [65]. OB-fold (the oligonucleotide/oligosaccharide-binding fold) is an ssDNA- or RNA-binding motif that binds PAR during DNA damage repair. OB-fold PAR-binding properties have been described for BRCA2 and SSB1 (with the *K_d_* 150–170 nM) [70,71]. Recently, KR-rich domains, SR repeats and RG/RGG-rich domains, have been shown to bind PAR, most likely because arginine and/or lysine enrichment are positively charged and bind PAR via electrostatic forces [72,73]. The structures of these domains, their abundance and PAR patterns to interact with, have been well described [69]. PAR-binding motifs have different binding characteristics and different *K_d_* of PAR. Some proteins have duplicated or triplicated binding domains that increase the stability of the complexes with PAR [74,75]. In addition to the difference in *K_d_* of motifs, structural features of motifs and diverse combinations of several motifs in a given PAR-binding protein, all motifs seem to determine the type of the cellular response in vivo.

### 3.3. PAR-Binding Proteins and Their Functions

The PARylation of targets is one of the important steps of DNA damage response (covalent and non-covalent binding; we will only talk non-covalent binders). Many PAR readers participate in DNA damage response (DDR). The function of PAR-binding by reader proteins during the DNA repair process can be divided into several steps: (a) fast and efficient recruitment of the DNA repair factors into the damaged sites, (b) DNA damage signal transduction, (c) apoptosis initiation, and (d) protein degradation. PAR synthesis and subsequent degradation by PARG are critical for DNA strand breaks repair [89].

A prominent and well documented PAR reader is XRCC1, which binds PAR with *K_d_* value 36 nM via the PBM domain within the BRCT domain (BRCA1 C-terminal). XRCC1 is recruited to DNA lesions by binding PAR chains longer than 7 units [90], required for base excision repair. Mutations LI360/361DD, W385D, and C389A in BRCT compromise the accumulation of XRCC1 at damaged sites [90,91]. On the other hand, XRCC1 binding to DNA lesions increases the amount of PAR formation, likely by sequestering PARG from the PARP1-PARG interaction [92]. However, the competition for PARP1 binding between XRCC1 and PARG seems to depend on the PAR: in instances of mild DNA damage PARG removes XRCC1 from PARP1 in the damaged foci [90], while after lethal doses of alkylating agent MNNG, XRCC1 disrupts PARG-PARP1 complexes to stimulate apoptosis [92]. The BRCT domain mediates the interaction of XRCC1 with PAR and DNA. The binding sites for PAR and DNA are different. PAR-binding defective double mutant R335A/K369A abolishes XRCC1 recruitment to damaged sites and shows a significant tail moment increase (U2OS cells). On the other hand, DNA-binding double mutant R399D/R400Q, which is able to bind PAR, shows slow recruitment to DNA damaged sites and thereby DNA repair defects [93].

The poly(ADP-ribose)-binding zinc finger (PBZ) motif are found in the checkpoint with FHA and RING finger (CHFR) and aprataxin- and PNK-like factor (APLF) [94]. Both proteins participate in DNA damage response and checkpoint regulation. CHFR is an E3 ubiquitin ligase and known to suppress the cell cycle progression [95]. It is recruited to damage sites by PAR and regulates the first wave of protein ubiquitination [96]. APLF belongs to the FHA (forkhead-associated) family of proteins, which is known to bind XRCC1 [74]. Accumulation of APLF at damaged sites also occurs through a strong PAR-binding (*K_d_* = 0.2 nM) via two PBZ domains and one FHA-domain. The tandem PBZ motifs significantly increase the stability of the complex (PAR-APLF) up to 0.95 nM. The FHA domain in the C-terminus of APLF decreases the *K_d_* for the APLF-PAR complex to 0.2 nM [76]. The recruitment of APLF to DNA damage sites and tight PAR-binding via its tandem PBZ motif can contribute to the regulation of the PAR intracellular level, because overexpression of APLF reduced PAR in early stages following DNA damage [89].

Breast cancer susceptibility gene 2 (BRCA2) is a key player in the homologous recombination repair of DSBs. BRCA2 contains three OB-folds which have been shown to bind DNA [97] and recruit exonuclease1 (EXO1) for strand resection. OB-folds of BRCA2 also bind PAR [71]. It is shown that the rapid recruitment (30 sec after the lesion) of BRCA2 to DNA lesions is initiated via the interaction with PAR, which however is much delayed in the presence of PARP inhibitor Olaparib [71]. The affinity between the OB-fold-PAR is very similar to OB-fold-ssDNA (*K_d_* are 100 nM and 200 nM respectively). The interaction of OB-fold-PAR thus likely serves to quickly load BRCA2 to DNA lesions [71].

EXO1 can also bind the PAR chain via the PIN domain (*K_d_* = 200 nM) [98]. Upon DNA damage, EXO1 begins to accumulate within 30 sec at the damaged sites determined by PIN-PAR- binding. Although the binding is not strong, it is obviously sufficient to recruit EXO1 to the DSB site [98]. At around 30 s, the kinetics of the recruitment are similar to BRCA2. Interestingly, BRCA2 depletion significantly decreases the speed of EXO1 recruitment [71], suggesting that PARylated BRCA2 facilitates EXO1 loading onto DNA breaks.

The cold-inducible RNA-binding protein (CIRBP) binds to 3′-UTR of stress-responsive and circadian clock RNAs and is found to participate in DSB repair via PAR-binding [99]. CIRBP is recruited to the DNA damage site and binds already existing PAR moieties, provided by autoPARylated PARP1, via its C-terminal RGG-rich motif—likely helped by its N-terminal RNA-recognition motif (RRM) [99]. PARylation mutant of all these residues in CIRBP blocks pS1981-ATM association with chromatin, but not phosphorylation level of ATM [99]. A similar effect has been observed for NBS1 and MRE11 accumulation on chromatin [99]. Thus, PARylated CIRBP can interact with ATM and the MRN complex to facilitate their binding to chromatin at the lesions.

PAR binders participate in the regulation of cell cycle progression, genome maintenance and transcription regulation [100,101]. Stalled replication forks activate PARP1, followed by PAR formation and S-phase checkpoint activation. We have shown a direct binding of the S-phase checkpoint kinase Chk1 with PAR via its PbR (PAR-binding regulation) motif, which is independent of ATR and its activity [77]. PbR motif of Chk1 belongs to PBZ domains, but has the non-canonical structure [77,102]. PAR deficiency attenuates Chk1 and phospho-Chk1 retention at stalled replication forks. PAR-binding to Chk1 stimulates Chk1 kinase activity. Mutations in the PbR-binding domain lead to hypersensitivity of cells to HU-induced cytotoxicity, mimicking the loss-of-function of Chk1 in response to stalled forks [77].

MacroH2A1.1, an isoform of macroH2A1 histone, is a well-known PAR reader [103]. Upon PARP1 activation, the macroH2A1.1 region loops toward PARylated proteins (for example, PARP1 and Ku70–Ku80). PARP1 inhibitor PJ-34 blocks microirradiation-induced chromatin rearrangements. Depending on the DNA damaging agent and amount of PAR (and probably structure), macroH2A1.1 influences chromatin rearrangement differently [103]. Thus, macroH2A1.1 macrodomain is sensitive to PARP1 activation and maintains chromatin plasticity in response to DNA damage [103].

E3 ubiquitin ligase Iduna (also known as RNF146) has both WWE (containing a PBM domain) and RING domains, both of which can bind PAR. The RING motif of Iduna stabilizes the binding of WWE to PAR [78]. The *K_d_* of WWE–RING–PAR interaction is ten times higher (around 40 nM) than that of WWE–PAR (around 400 nM) [75,104]. Separation of the WWE and RING domains leads to significant destabilization of the protein–PAR complex. The binding occurs via PAR clamping between the WWE/PBM and RING domains, which changes the conformation of RING and activates Iduna E3 ligase activity [104,105]. Binding of Iduna to PARylated proteins, especially autoPARylated PARP1, leads to degradation of PARylated proteins, which are important for certain cellular activities.

The WWE/PBM is also found in the apoptosis inducing factor (AIF). AIF interacts with PAR in a high affinity (*K_d_* is 66.3 nM) [106]. Upon binding to PAR, AIF is released from mitochondria and transported to the nucleus with subsequent parthanatos progression [106]. The decrease of PAR levels (for example, by PARG overexpression) inhibits AIF translocation to the nucleus. Iduna ubiquitinates and degrades AIF, thereby preventing cell death [107]. Iduna also binds Axin immediately after Axin PARylation by tankyrase, and ubiquitinates it, leading to activation of Wnt signaling [108,109]. Iduna acts on PARP1- and tankyrase-dependent PARylated proteins; whereas PARP1 modifies proteins with long and complex PAR chains, tankyrase adds short and low complexity of PAR to proteins. It is possible that Iduna can bind any PAR structure, but unclear how Iduna would choose the PARylated protein targets. One may assume that Iduna is able to clamp less branched and shortened PAR chains in the case of mild DNA damage, for example [110].

### 3.4. Length- and Branching-Dependent PAR-Binding

PAR chains are not homogenously linear ADPr polymers. Both ribose residues of the ADPr unit have the ability to link with the next ADPr [111]. Once PARP1 is activated it may form a PAR chain around 200 units long, with a branching period of every 20–50 units [112]. PARP1 is able to form the branched PAR structure, but PARP2 is thought to form mainly side chains of PAR. In PARP2-deficient cells the level of the branched PAR is two-fold lower [113]. PARP2 seems to be dependent on the PARP1 activity, because PARP2 accumulation in damaged sites is abrogated in PARP1^−/−^ MEFs [113]. PARP2 is activated by the interaction of its N-terminal region with the PAR chain formed by PARP1 and free nucleic acids (including DNA and RNA) in the cells. Activated PARP2 catalyzes additional PARylation on top of the existing PAR chains, followed by branched PAR chain formation [113].

The different characteristics of the polymer chain, such as the length and branched structure, influence the possibility of PAR-binding *K_d_* and consequently the cellular response. Several proteins are able to bind only long chains of PAR, others bind only the branched [113] or only the linear PAR chain [114]. Depending on the length and branch structure of PAR, PAR-binding proteins may have different characteristics. For example, the long PAR chain (55-mer) forms three specific complexes with p53, while the short chain (16-mer) forms only one complex type [115]. The strength of the interaction is dependent on the length of the chain (*K_d_* for 55-mer is 1.3 μM and *K_d_* for 16-mer is 25 μM). XPA forms a complex with 55-mer PAR chain, but not with ADPr shorter than 16-mer [115]. This effect has been observed similarly for chromatin regulator and DNA-binding protein DEK and H1 histone. DEK does not bind the PAR chain shorter than 34-mer, but the binding affinity increases in tandem with the increasing chain length. H1 binds the PAR chain 10-mer and more [116]. In addition, tandem PBZ motifs of APLF recognize branched PAR chain, while each PBZ motif can independently bind one or two ADPr residues [113]. Thus, different PAR readers react differently to the PAR structure. Table 3 lists major and defined PAR-binding motifs.

The variability of different PAR-binding proteins not only depends on different types of PAR-binding motifs, but also their binding characteristics. The interaction duration of different PAR-binding domains varies [98,108]. The type of interaction determines *K_d_* and influences the speed of the signaling process. One can hypothesize that different domains determine the type of PAR-mediated cellular responses. For example, both PBZ and PbR motifs represent Zinc finger type, but have only slight differences in the structure (Table 1), yet it is possible that they read different PAR patterns. PBZ is responsible for DNA damage and chromatin rearrangement [74,116], while PbR-PAR-binding starts cell cycle arrest after DNA damage [77]. Thus, the diversity of PAR structures and of PAR-binding motifs can dictate the function of PAR readers as (1) messengers from one activity or subcellular location to another, (2) modulators of target proteins, or (3) terminators of interactors and partners.

## 4. Perspectives

**Figure 4:**
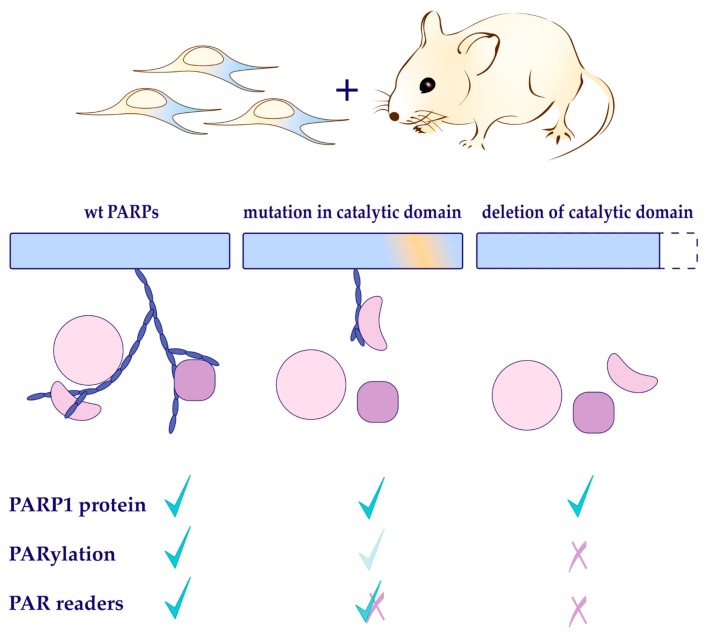
Genetic mutations of PARP1 in cells and mice have been used to study the function of PARP1 protein, PARylation activity, and PAR readers. While these studies have provided much insight into the function of PARP biology, it remains largely open as to how PARP1 protein, or its enzyme activity, or PAR readers are important in the cellular processes that dictate cell fate and pathological outcomes. Therefore, it calls for more defined and separation-of-function mutation studies.

PARP1 and PARylation play a multi-faceted function in many cellular processes, including DNA repair, genomic stability, chromatin remodeling, apoptosis, and aging, as well as in transcription regulation [8,45,131,132,133]. However, the biological function of PARP1 remains enigmatic. Several reasons can account for this: (1) PARP1 is a multi-domain containing proteins; (2) it is a NAD^+^-dependent enzyme whose activity is thought to be dependent mainly on its binding to DNA lesions, but recently also seems to be activated by chromatin conformation changes; (3) PARP1 produces a variety of PAR structures, which can be bound and recognized by diversity of proteins (PAR-readers); and (4) the affinity strength of PAR-binding by individual PAR-readers, the diversity of PAR-binding motifs of PAR readers, and the nature of PAR structures (linear vs. branched, long vs. short) can all influence the signaling (Figure 4).

PARP1 is an abundant cellular protein. Apart from its PARylation activity, the protein can serve as a scaffold protein to help other partners exert their function, for example in transcription activation. Due to space constraints, this review omits the discussion of potential PARP1 interaction partners. Despite huge effort, we are still far from fully understanding the biology of PARP1 and PARylation. For example, what function of PARP1 is dependent on its own protein structure (for example for DNA-binding, automodification, and interaction with its partners), or its enzymatic activity, or its product PAR or PAR readers (Figure 4)? To address these questions, we face challenges in biochemical techniques; for example, to synthesize defined PAR structures. There remains a missing link between protein scaffold function and its enzymatic activity. Advancing PARP research will provide insight into the function biology and biochemistry of PARylation-mediated cellular activities. Manipulation of enzymatic activities of PAR-synthetizing and -degrading proteins, such as PARPs and PARG, has been explored in many pharmacological interventions of pathologies and shows much promise for future treatment of human diseases, including cancer, diabetes, and stroke, as well as inflammation.

## Figures and Tables

**Table 1:** Important PARP mutants and the information achieved from their characterization.

Mutation	Enzymatic Activity	Amino Acid Role	Reference
K893R K893I	~40% ~0.2%	The initiation of the poly(ADP-ribosy1)ation reaction	[29]
D993E D993A	~15.2% ~0.2%	The initiation of the poly(ADP-ribosy1)ation reaction	[29]
K953R K953I	~2.9% ~9.8%	Indirect involvement in PARP activity	[29]
D914E D914A	~11.5% ~2.5%	Indirect involvement in PARP activity	[29]
E988Q E988A E988K	~2.2% 0.091% 1.25%	Key residues in the synthesis and elongation of PAR	[30,32]
L713F	~879%	Allosteric effect on the catalytic site	[31]
Y986S	11%	Enzymatic activity and PAR chain elongation	[32]
R847C E923G G972R	75% 20% 16%	PAR branching	[32]
C908R	<0.5%	Enzymatic activity	[32]
T316A W318R	~0.36% ~0.6%	Involvement in the DNA-dependent PARP1 activation	[24]
F44A V48A F44A/V48A	Lower auto-modification	DNA-binding affinity, DNA-dependent PARP-1 activation	[22]
Q40A D45A	Low auto-modification	Interactions with the domains essential for DNA-dependent activity	[22]
V144E/P149D V144E/P149I	ND	Recruitment towards the damage site	[25]
S499A/S507A/S519A	Low HPF1-dependent automodification	Automodification site, HPF1-dependent serine modification	[27]

**Table 2:** The list of PAR-binding motifs.

PAR-Binding Motif		Motif Structure	Described
**Zinc finger type**	PBZ	C_2_H_2_ type CX_5_CX_6_HX_5_H	[76]
	PbR	C_2_H_2_ type CX_8_CX_6_HX_8_H	[77]
	RING	C_3_HC_4_ type CX_2_CX_9-39_CX_1-3_HX_2-3_CX_2_CX_4-48_CX_2_C	[78]
Macrodomain		globular α/β/α sandwich β-α-β-α-α-β-β-α-β-α-β	[79]
PBM		[HKR]_1 × 2 × 3_[AIQVY]_4_[KR]_5_[KR]_6_[AILV]_7_ [FILPV]_8_	[78]
WWE		β2-β1-β6-β5-β4-β3 and/or β2-β1-β5-β3-β4	[80]
PIN-domain		Compact structure β1-α1-β2-α2-β3-α3-β4-α4-β5	[81]
FHA domain		Two β sheets with Greek key topology β2-β1-β11-β10-β7-β8 and β4-β3-β5-β6-β9	[82]
BRCT		β-α-β-β-α-β-α	[83]
OB-fold		Antiparallel β-barrel β1-β2-β3-β5-β4-β1	[84]
KR-rich domains, SR repeats, RG/RGG repeats		KR-, SR- or RG/RGG-rich repeats	[85,86,87]
RRM		[RK]_1_G_2_[FY]_3_[GA]_4_[FY]_5_V_6 × 7_[FY]_8_– X_n_–[LI]_1_[FY]_2_[VI]_3 × 4_[NG]_5_L_6_ β-α-β-β-α-β	[88]

**Table 3:** List of PAR binders.

PAR-Binding Motif	Example of Readers	Process	Reference
PBZ	APLF, CHFR	DNA damage, chromatin architecture	[74]
PbR	Chk1	DNA damage, cell cycle regulation	[77]
RING	RNF146/Iduna, Siah1, BARD1	DNA damage regulation, protein degradation, transcription.	[110,117,118]
Macrodomain	MacroH2A, PARG, TARG1, MacroD1, MacroD2, macroD3, ALC1, ARTD7, ARTD8, ARTD9, PARP9, PARP14, PARP15, GDAP2	DNA damage, redox defense, chromatin architecture, protein acetylation, viral infection	[79,119,120]
PBM	XRCC1, Aurora-A, NF-kappa-B, BID, CENP-A, ERCC-6, HKDC1, MVP, DNA topoisomerase 2-beta, BUB3, DNA ligase III, condensin complex subunit 1, hnRNP A1, hnRNP A2/B1, Ro(SS-A), H2A, H2B, H3, H4, AIF, MRE11, ATM, DNA-PKcs, KU70, MARCKS, MSH6, XPA, p21, DNA polymerase epsilon, NOS2, CAD, TERT, CTCF, DNMT1, Par6, DEK, WRN, HK1	DNA damage, immune response, cell cycle regulation, chromatin architecture, telomeres length, stress signaling	[116,121,122,123,124]
WWE	RNF146/Iduna, PARP11, PARP13, PARP14, Deltex1 (A and B), Deltex2 (A and B), Deltex4 (A and B), ULF, HUWE1, DDHD2	DNA damage regulation, protein degradation, mRNA stability	[75,110,125,126]
PIN-domain	EXO1, GEN1, SMG5	DNA damage	[98]
FHA domain	APLF, PNKP, APTX	DNA damage	[74,127]
BRCT	BARD1, APLF, Ligase4, XRCC1, NBS1	DNA damage	[74,118,127]
OB-fold	BRCA2, SSB1, SSB2, CTC1, MEIOB	DNA damage	[70,71]
KR-rich domains, SR repeats, RG/RGG repeats	G3BP, ASF/SF2, CHD6, MTCL1, dMi-2, CIRBP, FUS/TLS, TAF15, EWS	DNA damage, chromatin architecture, stress response, transcription and RNA processing	[72,73,85,86,87,99,128,129]
RRM	ASF/SF2, CIRBP, FUS/TLS, TAF15, EWS, NONO	DNA damage, RNA processing	[85,87,99,129,130]
